# Accurate and sensitive detection of *Salmonella* in foods by engineered bacteriophages

**DOI:** 10.1038/s41598-020-74587-8

**Published:** 2020-10-15

**Authors:** Minh M. Nguyen, Jose Gil, Matthew Brown, Eduardo Cesar Tondo, Nathanyelle Soraya Martins de Aquino, Marcia Eisenberg, Stephen Erickson

**Affiliations:** 1 Laboratory Corporation of America Holdings, New Brighton, MN 55112 USA; 2 Laboratory Corporation of America Holdings, Los Angeles, CA 90062 USA; 3 Laboratory Corporation of America Holdings, Burlington, NC 27215 USA; 4 Laboratório de Microbiologia e Controle de Alimentos, Instituto de Ciência e Tecnologia de Alimentos, Universidade Federal do Rio Grande do Sul (ICTA/UFRGS), Porto Alegre, RS 91501-970 Brazil

**Keywords:** Bacteriophages, Food microbiology

## Abstract

*Salmonella* is a major causative agent of foodborne illness and rapid identification of this pathogen is essential to prevent disease. Currently most assays require high bacterial burdens or prolonged enrichment to achieve acceptable performance. A reduction in testing time without loss of sensitivity is critical to allow food processors to safely decrease product holding time. To meet this need, a method was developed to detect *Salmonella* using luciferase reporter bacteriophages. Bacteriophages were engineered to express NanoLuc, a novel optimized luciferase originating from the deep-sea shrimp *Oplophorus gracilirostris*. NanoLuc-expressing bacteriophages had a limit of detection of 10–100 CFU per mL in culture without enrichment. Luciferase reporters demonstrated a broad host range covering all *Salmonella* species with one reporter detecting 99.3% of 269 inclusivity strains. Cross-reactivity was limited and only observed with other members of the *Enterobacteriaceae* family. In food matrix studies, a cocktail of engineered bacteriophages accurately detected 1 CFU in either 25 g of ground turkey with a 7 h enrichment or 100 g of powdered infant formula with a 16 h enrichment. Use of the NanoLuc reporter assay described herein resulted in a considerable reduction in enrichment time without a loss of sensitivity.

## Introduction

*Salmonella* is one of the most common foodborne pathogens resulting in over 93 million cases of salmonellosis and 150,000 deaths every year globally^1^. Within the US, it is estimated to cause over a million infections annually and is the leading cause of hospitalizations and deaths from foodborne illnesses^2^. These infections also represent a substantial economic burden with an annual cost of illness estimated at over $3 billion in the US alone^3^.

The genus *Salmonella* consists of two species: *enterica* and *bongori*. *Salmonella enterica* is further divided into six taxonomically recognized subspecies: *enterica*, *salamae*, *arizonae*, *diarizonae*, *houtenae*, and *indica*^4^. Critically, 99% of *Salmonella* isolates from human cases in the US are *Salmonella enterica* subspecies *enterica*^5^. This subspecies can be further differentiated into over 1500 serovars^6^. The most prevalent serovars associated with foodborne disease outbreaks in the US are Enteritidis (32% of outbreaks), Typhimurium (13%), Heidelberg (8%) and Newport (7%)^7^.

Accurate and timely detection of contaminated food prior to sale is essential in preventing foodborne illness. The current gold standard method for *Salmonella* detection requires at least three days, consisting of multiple sample enrichments and subsequent plating on selective agar^8,9^. An additional 24 h is also required, at minimum, to confirm any presumptive positives identified, traditionally using biochemical analysis. Although laborious, this method can detect a single *Salmonella* colony forming unit (CFU) in a 25-g sample.

A reduction in total testing time is highly desirable and can be achieved by a decrease in detection and/or enrichment time. Assays using PCR, ELISA, latex agglutination, mass spectrometry, and even meta-genomic sequencing, have been explored as rapid alternative methods of *Salmonella* detection in food matrices^10–14^. Additionally, capture of *Salmonella* by antibodies, DNA aptamers, or bacteriophages has been used to concentrate samples and reduce traditional enrichment times^15–17^. While these rapid approaches have been largely successful, most available methods still require at least 18 h of enrichment to detect 1 CFU in 25 g of product.

Bacteriophages (phages) have also been examined as a foundation for sensitive and accurate detection of foodborne pathogens^18,19^*.* One particularly promising phage-based approach for *Salmonella* detection involves the use of luciferase reporter phages^20–22^. This method requires an engineered phage, traditionally encoding the luciferase gene cassette *lux* from *Allivibrio fischeri*. If a sample contains viable contaminating *Salmonella*, infection with the recombinant phage will yield a detectable bioluminescent signal. A novel luciferase, NanoLuc, has been recently engineered from the deep-sea shrimp *Oplophorus gracilirostris*^23^. This luciferase is only 19 kDa, 150 times brighter than other luciferases and reacts with a novel furimazine substrate with low background noise^24^. These characteristics suggest that NanoLuc would be a superior choice as a luciferase reporter in phage-based assays. Although yet to be achieved in *Salmonella*, NanoLuc reporter phages have been recently described mediating sensitive and rapid detection of *Escherichia coli* O157:H7 or *E. coli* in ground beef and water^25–27^.

The objectives of this study were: (1) to develop and characterize the first NanoLuc reporter phage assay for *Salmonella* and (2) to assess its performance to detect this pathogen in ground turkey and powdered infant formula (PIF).

## Results

### Characterization of *Salmonella* phages SEA1 and TSP1

Preliminary studies led to the selection of two lytic *Salmonella* bacteriophages, SEA1 and TSP1, for assay development. Using one-step growth curves, the replication cycle time was determined to be 35–40 min and 60–70 min for SEA1 and TSP1, respectively. The burst size of SEA1 was found to be approximately 30 pfu per cell, while TSP1 produced a larger burst size of approximately 100 pfu per cell. Thus, the replication cycle time and burst size for SEA1 and TSP1 are similar to those reported for other *Salmonella* phages^28^.

**Figure 1:**
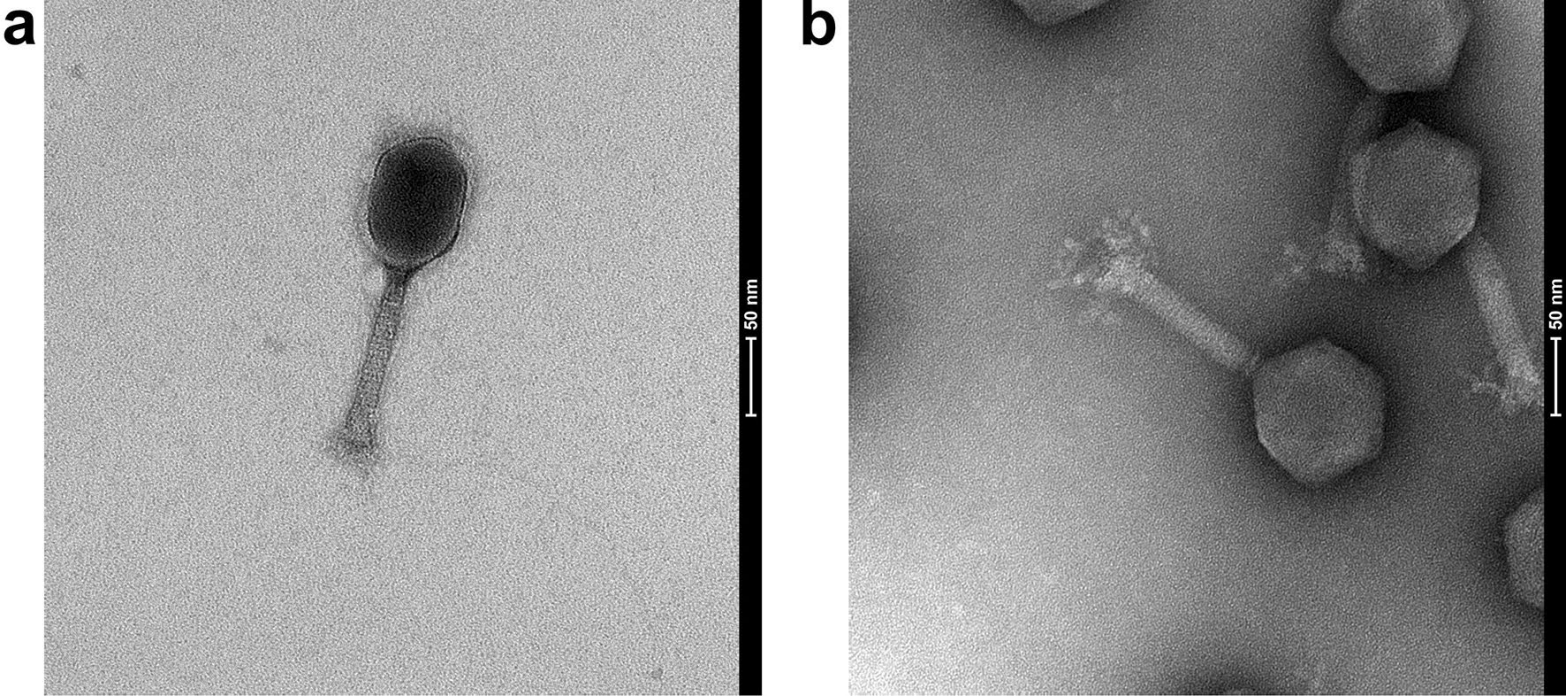
Transmission electron micrograph of (**a**) SEA1; (**b**) TSP1.

To facilitate plasmid design for homologous recombination, DNA from SEA1 and TSP1 was extracted and sequenced. While genome curation and annotation are beyond the scope of this study, preliminary analysis revealed a genome size of approximately 162 kbp for SEA1 and 157 kbp for TSP1. BLAST analysis of SEA1 revealed considerable homology to *Salmonella* phage vB_SenM-S16 (NC_020416). This phage was previously described as a 160 kbp Myovirus possessing a remarkably broad host range strictly within the *Salmonella* genus^29^. BLAST analysis of TSP1 revealed considerable homology to *Salmonella* phage SFP10 (NC_016073). This phage was previously described as a 158 kbp Myovirus specific for *E. coli* O157:H7 and *Salmonella* isolates^30^. The morphology of SEA1 and TSP1 was visualized by transmission electron microscopy (Fig. 1a,b). Both SEA1 and TSP1 have contractile non-flexible tails. Based upon these micrographs and supported by sequence homology, SEA1 and TSP1 are also predicted members of the *Myoviridae* family^31^.

### Construction of NanoLuc-expressing recombinant bacteriophages

**Figure 2:**
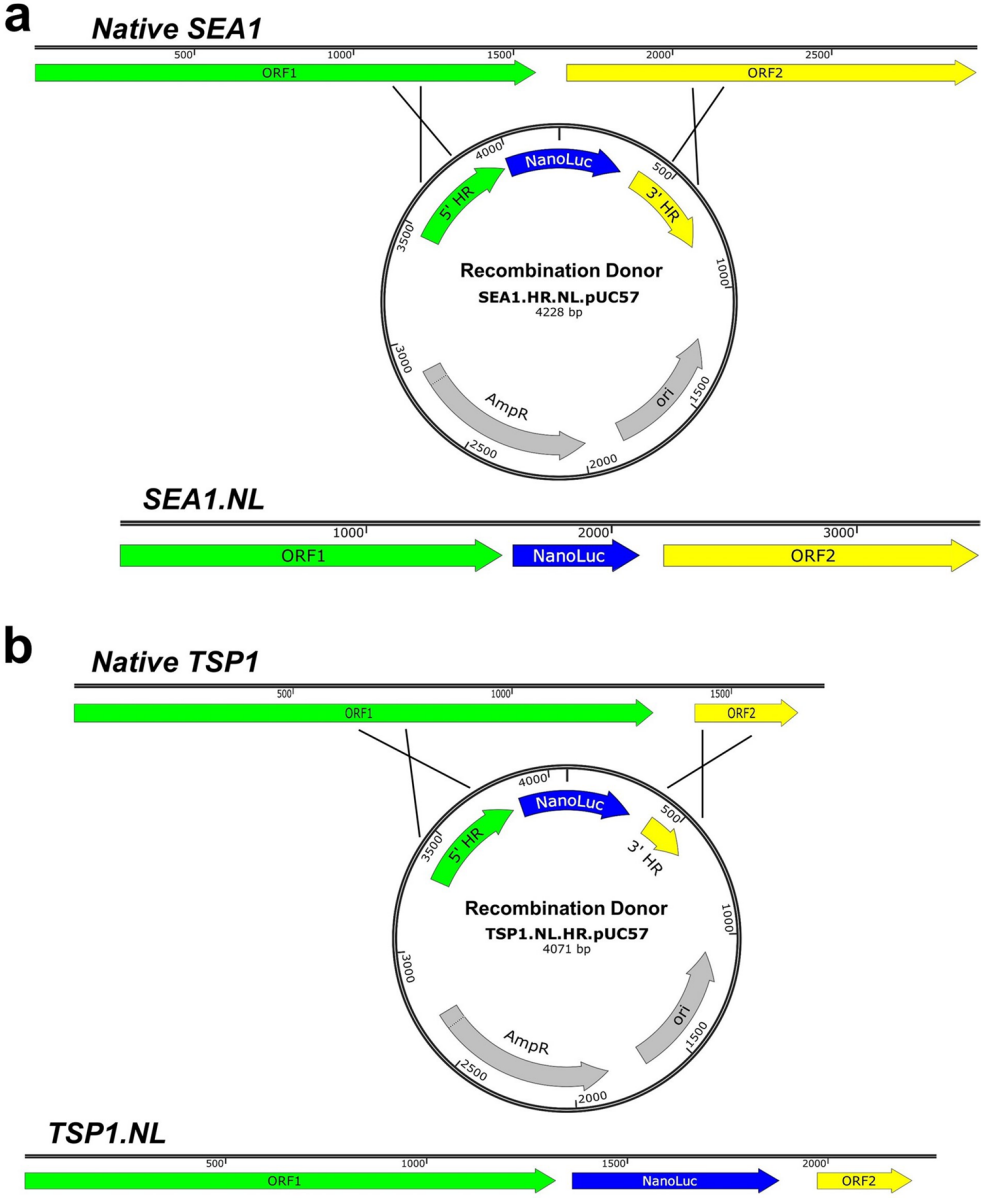
Generation of NanoLuc expressing bacteriophages by homologous recombination. (**a**) SEA1 recombination donor assembled in pUC57. ORF1 of SEA1 indicates the predicted major capsid protein while ORF2 has homology to head vertex proteins; (**b**) the TSP1 recombination donor assembled in pUC57. ORF1 of TSP1 indicates the predicted major capsid protein while ORF2 is a hypothetical protein of unknown function. Graphics were generated using SnapGene (GSL Biotech LLC, Chicago, IL, USA).

Generation of NanoLuc-expressing recombinant SEA1 and TSP1 was performed using homologous recombination (Fig. 2a,b). Homologous flanks were designed to direct insertion downstream of the predicted major capsid protein, a strategy previously used to generate luciferase reporter phage for *Listeria*^32^. This insertion site was not expected to disrupt any predicted genes. Recombination donor plasmids were generated containing these regions of homology flanking a codon-optimized NanoLuc gene under a T4 late promoter. *Salmonella* transformants containing these donor plasmids were infected with SEA1 and TSP1. Recombinant NanoLuc-expressing bacteriophages (SEA1.NL and TSP1.NL) were isolated from this reaction, passaged to purity, and confirmed by DNA sequencing.

### Limit of detection of engineered bacteriophage reporters

Successful detection of pathogens must be capable of finding small numbers of contaminating cells. Although SEA1.NL and TSP1.NL were expected to produce considerable amounts of luciferase following infection, the exact number of required host cells to produce a detectable signal over background was unknown. To determine the limit of detection of these engineered phages, infections were performed with inoculums ranging from 1 to 10,000 CFU. After a 2 h infection, the amount of luciferase produced in each condition was determined with a luminometer following the addition of substrate (Table 1). Background from medium and reporter alone (negative control) was minimal, averaging 117 and 51 RLU for SEA1.NL and TSP1.NL, respectively. The standard deviation (SD) of background was also low, allowing signal from luciferase production to be easily recognized. Signal above background was detected, on average, from a single cell for both SEA1.NL and TSP1.NL. Average RLU from either reporter phage infection increased proportionately with the number of *Salmonella* cells, reaching values over 100 times background with only 100 CFU. Signal from TSP1.NL was consistently higher than SEA1.NL at equivalent cell counts, possibly reflective of the higher burst size of TSP1. RLU variability, as measured by coefficient of variation (CV), was expected at low cell counts, where the probability of no cells being present in a replicate is increased. Individual replicates that received no cells by chance will yield only a background signal, which will contrast starkly with replicates containing live cells. This effect would be most pronounced at one, two, and five CFU, where the highest variation is observed. These results confirm the functionality of SEA1.NL and TSP1.NL, revealing a clear correlation between average RLU and presence of *Salmonella*. Remarkably, a detectable signal above background could be demonstrated with a single log phase CFU after only a 2 h infection. This single cell signal was greater than twice background, a standard cutoff used by others to determine positive detection from luciferase reporter phages^25^.

**Table 1:** Limit of detection of bacteriophages SEA1.NL and TSP1.NL.

Reporter	CFU	# of replicates	Avg. RLU	SD	% CV	S/B
SEA1.NL	0	6	117	7	6	1.0
	1	10	287	499	174	2.4
	2	10	365	382	105	3.1
	5	10	1285	1172	91	11.0
	10	10	2205	960	44	18.8
	100	10	12,453	4685	38	106.4
	1000	6	169,643	26,610	16	1449.9
	10,000	6	2,313,504	223,614	10	19,773.5
TSP1.NL	0	6	51	12	24	1.0
	1	10	207	255	123	4.1
	2	10	362	497	137	7.1
	5	10	627	704	112	12.3
	10	10	2667	2163	81	52.1
	100	10	20,920	5011	24	408.9
	1000	6	241,224	19,632	8	4714.5
	10,000	6	4,585,851	144,389	3	89,625.8

*Salmonella enterica* subsp. *enterica* ser. Typhimurium (ATCC 19585) was used for TSP1.NL while *Salmonella enterica* subsp. *enterica* ser. Choleraesuis (ATCC 7001) was used for SEA1.NL. Strains were diluted from log phase cultures and infected with the indicated reporter phage for 2 h. Signal over background was defined as average RLU over average RLU without cells.

*CFU* colony forming units, *RLU* relative light units, *SD* standard deviation, *CV* coefficient of variation, and *S/B* signal over background.

### Inclusivity of SEA1NanoLuc and TSP1NanoLuc detection

Since most human infections are the result of *Salmonella enterica* subsp. *enterica*, a panel of 245 members of this subspecies was assembled. These strains, spanning 84 distinct serovars, were infected for 2 h with either SEA1.NL or TSP1.NL and assessed for luciferase production. A threshold of 750 RLU was used to establish positive detection for inclusivity and all further testing. This static value was selected for increased stringency during host range testing and to allow consistency across complex matrices with variable background autoluminescence. When testing stationary phase cells without enrichment, SEA1.NL and TSP1.NL produced a positive signal from 243 of 245 and 129 of 245 *Salmonella enterica* subsp. *enterica* strains, respectively (Table 2). Data for individual serovars are provided (Supplementary Table 1). Only two *Salmonella* strains were negative with both reporter phages, one strain of serovar Enteritidis (out of 27 tested) and one strain of serovar Kentucky (out of 3 tested). Although less common, *Salmonella bongori* and *Salmonella enterica* subspp. *arizonae, diarizonae, houtenae, indica* and *salamae* may facilitate human disease and cannot be disregarded. Additional 24 *Salmonella* strains were assessed, including at least one representative of every currently recognized *Salmonella enterica* subspecies and the only other *Salmonella* species, *bongori*. SEA1.NL produced a positive signal from all 24 strains, while TSP1.NL detected six of these strains (Table 2). RLU values for all inclusivity strains are provided (Supplementary Table 2). Overall, SEA1.NL yielded an impressively broad host range, successfully detecting 99.3% of *Salmonella* tested in this study. TSP1.NL, on the other hand, detected just over one half (50.2%) of strains tested, indicating a substantially narrower host range. Of interest, TSP1.NL produced substantially higher signal than SEA1.NL for the Agona serovar of *Salmonella enterica* subsp. *enterica*. With six strains of this serovar tested, the median RLU signal was 73,530 for SEA1.NL and 51,280,951 for TSP1.NL (Supplementary Table 2). While no false negatives were observed with these six strains, the lowest signal observed with SEA1.NL was a mere 2958 RLU compared to 6,195,000 RLU with TSP1.NL. This particular serovar has been the source of several food-related outbreaks, including contaminated infant milk products^33–35^. Overall, the median signal from positively detected *Salmonella* strains was 103,257,000 RLU for SEA1.NL and 206,576,768 RLU for TSP1.NL. These values are substantially above the 750 RLU threshold used and demonstrate the robust signal generation of these reporters.

**Table 2:** Detection of diverse *Salmonella* by SEA1.NL and TSP1.NL.

Genus	Species	Subspecies	Positives/total	
			SEA1.NL	TSP1.NL
*Salmonella*	*enterica*	*enterica*	243/245	129/245
		*salamae*	6/6	4/6
		*arizonae*	6/6	1/6
		*diarizonae*	6/6	1/6
		*houtenae*	2/2	0/2
		*indica*	1/1	0/1
	*bongori*	N/A	1/1	0/1
	Non-typeable	N/A	2/2	0/2
Summary			267/269	135/269

Stationary phase cultures were diluted to an OD_600_ of 0.2 and infected with the indicated reporter phage for 2 h. Strains were determined to be positive when signal exceeded a detection threshold of 750 RLU. Strains were determined to be non-typeable by vendor/source.

### Specificity of SEA1NanoLuc and TSP1NanoLuc detection

Methods facilitating detection of *Salmonella* contamination must possess sufficient specificity to limit cross-reactivity with the natural microbiome present in many food matrices. To determine the specificity of these NanoLuc reporter phages, an exclusivity panel of non-*Salmonella* strains was collected. Representatives of 14 species of Gram-positive and 26 species of Gram-negative bacteria were infected for 2 h with SEA1.NL or TSP1.NL and assessed for luciferase production. Unsurprisingly, no false positives were detected with Gram-positive bacteria, likely due to considerable differences in surface structures when compared to the *Salmonella* host (Table 3). False positives were observed, however, with several Gram-negative species. Upon infection of diluted overnight cultures with SEA1.NL, a total of eight false positives were identified from the panel of 90 strains. For this reporter, three strains of *Escherichia coli* (of 48 tested) and one strain each of *Citrobacter brakii*, *Citrobacter sedlakii*, *Serratia marcescens*, *Shigella flexneri*, and *Yersinia enterocolitica* produced a signal above the detection threshold. By contrast, TSP1.NL only produced a positive signal with one strain, a *Citrobacter sedlakii*, from the entire exclusivity panel. RLU values for all exclusivity strains are provided (Supplementary Table 3). Of note, five of the eight false positives encountered with SEA1.NL were below 103,000 RLU, which is 1000-fold lower than the median RLU signal observed with *Salmonella* true positives. Only two false positive strains, one *Escherichia coli* and one *Citrobacter sedlakii* generated signal indistinguishable from most of the *Salmonella* strains. These results highlight the limited potential for false positives from related Gram-negative bacteria.

**Table 3:** Exclusivity of SEA1.NL and TSP1.NL.

Type	Genus	Species	Positive/total	
			SEA1.NL	TSP1.NL
Gram-negative	*Acinetobacter*	*calcoaceticus*	0/1	0/1
	*Citrobacter*	*braakii*	1/1	0/1
		*freundii*	0/1	0/1
		*koseri*	0/1	0/1
		*sedlakii*	1/1	1/1
		*werkmanii*	0/1	0/1
		*youngae*	0/1	0/1
	*Cronobacter*	*sakazakii*	0/1	0/1
	*Edwardsiella*	*tarda*	0/1	0/1
	*Enterobacter*	*cloacae*	0/1	0/1
		*kobei*	0/1	0/1
	*Escherichia*	*coli*	3/48	0/19
		*fergusonii*	0/1	0/1
		*hermanni*	0/1	0/1
	*Hafnia*	*alevi*	0/1	0/1
	*Klebsiella*	*aerogenes*	0/1	0/1
		*oxytoca*	0/1	0/1
		*pneumonia*	0/1	0/1
	*Morganella*	*morganii*	0/1	0/1
	*Pluralibacter*	*gergoviae*	0/1	0/1
	*Proteus*	*vulgaris*	0/1	0/1
	*Pseudomonas*	*aeruginosa*	0/1	0/1
	*Serratia*	*marcescens*	1/1	0/1
	*Shigella*	*flexneri*	1/1	0/1
		*sonnei*	0/1	0/1
	*Yersinia*	*enterocolitica*	1/1	0/1
Gram-positive	*Bacillus*	*cereus*	0/1	0/1
		*subtilis*	0/1	0/1
	*Enterococcus*	*faecalis*	0/2	0/2
		*faecium*	0/1	0/1
	*Listeria*	*grayi*	0/1	0/1
		*innocua*	0/1	0/1
		*ivanovii*	0/1	0/1
		*seeligeri*	0/1	0/1
		*welshimeri*	0/1	0/1
	*Staphylococcus*	*aureus*	0/3	0/3
		*epidermidis*	0/1	0/1
		*haemolyticus*	0/1	0/1
		*hominis*	0/1	0/1
		*saprophyticus*	0/1	0/1
Summary			8/90	1/61

Stationary phase cultures were diluted to an OD_600_ of 0.2 and infected for 2 h with the indicated reporter phage. Samples were positive when the luminescent signal exceeded a threshold of 750 RLU.

### Detection of *Salmonella* contamination in food matrices

Based upon these results, SEA1.NL and TSP1.NL were combined into a single phage cocktail. TSP1.NL was expected to supplement the signal intensity of many *Salmonella* strains without compromising the specificity achieved with SEA1.NL alone. Further, an enrichment period was added to the workflow to promote recovery, growth, and detection of single cell *Salmonella* contamination. This method of phage detection is referred to as the PhageDx method for *Salmonella*.

Although accurate detection of *Salmonella* cells was achieved in pure culture, food products are complex test matrices that may present additional challenges and complications. To determine if the PhageDx method could mediate detection in such environments, two distinct relevant food matrices were selected. As a representative of a ground meat product, raw ground turkey was chosen and has previously been associated with a nationwide outbreak in the United States^36^. For the second matrix, powdered infant formula was selected to model detection in dried food products, which itself has been linked to multiple outbreaks in infants^37^. Both matrices were pre-screened prior to use to evaluate the presence of pre-existing contamination. *Salmonella* was not detected endogenously from portions of either matrix used in this study. Although appearing free of *Salmonella*, each gram of homogenized ground turkey did yield approximately 40 CFU on non-selective media. The powdered infant formula used in this study was found to contain little endogenous flora (0 CFU per gram).

Portions (25 g) of raw ground turkey were either uninoculated, inoculated with a low-level of *Salmonella* (~ 1 CFU), or inoculated with a high-level of *Salmonella* (~ 10 CFU). Following a 7 h enrichment, samples were infected with a reporter phage cocktail for 2 h and checked for luciferase production. No false positives were detected among uninoculated test portions (Table 4). As anticipated, partial positives for the “low” inoculum and all positives for the “high” inoculum were obtained with two *Salmonella enterica* subsp. *enterica* serovars. Importantly, the PhageDx assay agreed with a culture-based confirmation method for all 30 samples. Dynabead isolation and CHROMagar *Salmonella* were utilized for this comparison as both methods have demonstrated excellent performance in a variety of food matrices^38–41^. These results indicate that the PhageDx method is capable of accurately detecting low levels of *Salmonella* contamination in raw ground turkey.

**Table 4:** Detection of *Salmonella enterica* in inoculated portions of ground turkey (25 g).

Serovar	Inoculum	RLU	PhageDx	Culture
Newport	None	230	Negative	Negative
	None	269	Negative	Negative
	None	308	Negative	Negative
	Low	227	Negative	Negative
	Low	280	Negative	Negative
	Low	283	Negative	Negative
	Low	15,287	Positive	Positive
	Low	228,509	Positive	Positive
	Low	241,967	Positive	Positive
	Low	258,108	Positive	Positive
	Low	379,592	Positive	Positive
	Low	496,149	Positive	Positive
	Low	921,481	Positive	Positive
	High	5,037,793	Positive	Positive
	High	5,060,438	Positive	Positive
Muenster	None	203	Negative	Negative
	None	203	Negative	Negative
	Low	168	Negative	Negative
	Low	207	Negative	Negative
	Low	209	Negative	Negative
	Low	219	Negative	Negative
	Low	223	Negative	Negative
	Low	244	Negative	Negative
	Low	14,893	Positive	Positive
	Low	76,050	Positive	Positive
	Low	347,209	Positive	Positive
	Low	690,403	Positive	Positive
	High	1,065,705	Positive	Positive
	High	1,834,854	Positive	Positive
	High	2,683,763	Positive	Positive

*Salmonella enterica* subsp. *enterica* serovars were diluted from stationary phase cultures and inoculated into pre-screened portions of ground turkey. Strain 27869 (ATCC) and OCT084 (USDA) were used as serovar Newport and Muenster, respectively. Equilibrated samples were enriched for 7 h, and infected with a cocktail of SEA1.NL and TSP1.NL for 2 h before being assessed for luciferase production. Inoculums consisted of either no CFU “None”, approximately 1 CFU per 25 g “Low” (1.1 CFU for Newport, 1.2 CFU for Muenster), or approximately 10 CFU per 25 g “High” (11.8 CFU for Newport, 8.9 CFU for Muenster). A detection threshold of 750 RLU was used to determine positive samples. Samples were confirmed by a culture-based method involving Dynabead isolation and plating on CHROMagar *Salmonella.*

Portions (100 g) of PIF were evaluated in a similar manner to raw ground turkey, except a 16 h enrichment was used. No false positives were detected among uninoculated test portions (Table 5). Both serovars of *Salmonella enterica* subsp. *enterica* produced the anticipated partial positives for the “low” inoculum and all positives for the “high” inoculum. As seen previously with ground turkey, the PhageDx method agreed with a culture-based confirmation method for all 30 PIF samples. These results indicate that the PhageDx method is also capable of accurately detecting low levels of *Salmonella* contamination in PIF.

**Table 5:** Detection of *Salmonella enterica* in inoculated portions of powdered infant formula (100 g).

Serovar	Inoculum	RLU	PhageDx	Culture
Heidelberg	None	339	Negative	Negative
	None	354	Negative	Negative
	None	376	Negative	Negative
	Low	285	Negative	Negative
	Low	288	Negative	Negative
	Low	333	Negative	Negative
	Low	343	Negative	Negative
	Low	395	Negative	Negative
	Low	432	Negative	Negative
	Low	461	Negative	Negative
	Low	259,893,152	Positive	Positive
	Low	461,765,216	Positive	Positive
	Low	501,775,552	Positive	Positive
	High	498,655,520	Positive	Positive
	High	1,031,197,312	Positive	Positive
Reading	None	271	Negative	Negative
	None	305	Negative	Negative
	Low	302	Negative	Negative
	Low	334	Negative	Negative
	Low	2820	Positive	Positive
	Low	10,667	Positive	Positive
	Low	16,944	Positive	Positive
	Low	43,975	Positive	Positive
	Low	162,731	Positive	Positive
	Low	167,912	Positive	Positive
	Low	458,206	Positive	Positive
	Low	481,718	Positive	Positive
	High	170,377	Positive	Positive
	High	340,074	Positive	Positive
	High	365,167	Positive	Positive

*Salmonella enterica* subsp. *enterica* serovars were diluted from stationary phase cultures, dried down, and inoculated into pre-screened portions of PIF. Strain SL476 (FDA) and 52317.1 (USDA) were used as serovar Heidelberg and Reading, respectively. Equilibrated samples were enriched for 16 h, diluted ten-fold, and infected with a cocktail of SEA1.NL and TSP1.NL for 2 h before being assessed for luciferase production. Inoculum consisted of either no CFU “None”, 1 CFU per 100 g “Low”, or 5 CFU per 100 g “High”. A detection threshold of 750 RLU was used to determine positive samples. Samples were confirmed by a culture-based method involving plating on CHROMagar *Salmonella.*

## Discussion

Rapid, accurate, and sensitive detection of foodborne pathogens is essential to maintain a safe and effective food supply. Despite achieving desired sensitivity and accuracy, many commercially available assays require extensive enrichment and operate under a timeframe of days, not hours. This study details the development and performance of the PhageDx rapid detection method for *Salmonella*, a prevalent and important contaminant of food products. To our knowledge, this method represents the first development and use of NanoLuc reporter bacteriophages in *Salmonella*.

Initial screening of 53 bacteriophages led to two promising candidates, SEA1 and TSP1, being chosen for assay development. These phages were predicted based on morphology to be members of the *Myoviridae* family (Fig. 1). NanoLuc-expressing recombinants of these phages (SEA1.NL and TSP1.NL) were engineered using homologous recombination (Fig. 2). These reporters were evaluated using a 2 h infection, no enrichment, and varying amounts of log phase *Salmonella*. A clear correlation between luminescent signal over background and *Salmonella* was observed with SEA1.NL and TSP1.NL (Table 1). Critically, a single CFU of *Salmonella* produced, on average, a detectable signal above background. This result underscores the advantages of tying reporter production to lytic phage, which rapidly adsorb, infect, replicate, and lyse host cells. The limit of detection of this method is also aided by the robust and specific luminescent signal produced by NanoLuc and its substrate, furimazine^23^. The reporter phages engineered in this study thus demonstrated remarkable sensitivity, a promising trait that can be leveraged to shorten assay time.

The inclusivity of NanoLuc reporter phages was evaluated with diluted stationary phase cultures, no enrichment, and a 2 h infection. Using a detection threshold of 750 RLU, SEA1.NL was able to positively detect 99.3% of 269 *Salmonella* strains (Table 2). The extensive range of this reporter suggests that SEA1 utilizes a receptor common to almost all *Salmonella*. Of note, the *Salmonella* bacteriophage S16, which has considerable homology to SEA1, has been found to bind OmpC, an outer member protein that is well-conserved among *Salmonella*^29,42^. Regardless of the receptor used, the median signal from detected strains was over 100 million RLU, well beyond the threshold used (Supplementary Table 2). Unlike the broad coverage of SEA1.NL, TSP1.NL detected only 50.2% of these strains while retaining strong median signal over 200 million RLU among positives. The narrower host range of TSP1 may represent the utilization of a less common receptor. Possible mechanisms include various outer membrane proteins or LPS O-antigen modifications found to act as receptors in other *Salmonella* phage^43^. Overall, the broad inclusivity of SEA1.NL is well-suited for detection across the *Salmonella* genus, while TSP1.NL may provide a supportive benefit with signal intensity in particular serovars, such as Agona.

Only two strains of *Salmonella* could not be detected by either reporter, one representative each of *Salmonella enterica* subsp. *enterica* serovars Enteritidis and Kentucky. Importantly, our results do not indicate an inability to detect these serovars entirely as 27 other Enteritidis and two other Kentucky strains were positive (Supplementary Table 1). The exact mechanism behind these two false negatives is unknown, although it is plausible these two strains lack a common *Salmonella* receptor. Alternatively, a litany of phage resistance mechanisms have been described such as those mediating restriction-modification, abortive infection, and extracellular matrix production^44^. Future studies may refine or expand upon the phage cocktail, highlighting the flexible and modular nature of this approach.

The exclusivity of each reporter phage was evaluated in the same fashion as inclusivity. Of 90 non-*Salmonella* strains examined, false positives were detected with eight strains for SEA1.NL (Table 3). These eight strains belonged to the family *Enterobacteriaceae* with representatives of five genera including *Escherichia*, *Citrobacter*, *Serratia*, *Shigella*, and *Yersinia*. It appears likely that the receptor of SEA1, while conserved among most *Salmonella*, is not restricted to this genus. Some outer membrane proteins, including OmpC, are conserved among members of this family and may participate in this cross-reactivity^45–47^.

Among the eight false positives, only one strain of *Citrobacter sedlakii* and one strain of *Escherichia coli* could mimic the intensity of a typical *Salmonella* isolate (Supplementary Table 3). Although the mechanism behind this variation in signal intensity is not known, it is feasible that non-*Salmonella* may lack certain co-receptors conducive to phage infection. TSP1.NL, on the other hand, cross-reacted with only one strain from the entire exclusivity panel, the same *Citrobacter sedlakii* observed with SEA1.NL. This supports the previous notion regarding the narrower host range of this phage. TSP1.NL has considerable homology to the *Salmonella* bacteriophage SFP10, which was previously reported to recognize both *Salmonella* and *E. coli* O157:H7^30^. Despite this homology, TSP1.NL did not produce a positive result for any O157:H7 strain tested in our study, which included some of the same strains previously tested with SFP10. These data support the notion that TSP1.NL does not share this property with SFP10.

The performance of SEA1.NL and TSP1.NL in food matrices was evaluated as part of the PhageDx *Salmonella* assay. Critically, this method involves minimal processing, no sample cleanup, and can be adapted as needed depending on the properties of the matrix. When compared to a culture-based method, the PhageDx assay correctly identified 100% of artificially contaminated raw ground turkey samples with a 7 h enrichment (Table 4). Detection of a single *Salmonella* CFU in 25 g of product, a required sensitivity benchmark, was achieved. Importantly, no false positives were observed, suggesting that the natural flora of this matrix (40 CFU per g) did not contain problematic *Enterobacteriaceae* strains or burdens. Similar performance was achieved in PIF, where the PhageDx assay also correctly identified 100% of artificially contaminated PIF samples with a 16 h enrichment (Table 5). The lack of false positives in this matrix was unsurprising, given the lack of natural flora observed in this study (0 CFU per g). The ability of phage reporters to function in various food matrices has been previously observed and is further supported by this study^48,49^. In summary, the PhageDx method agreed 100% with the longer culture-based method in both food matrices and was capable of accurate and sensitive detection of *Salmonella* contamination.

Previous attempts to utilize bacteriophage for *Salmonella* detection in food matrices have been met with mixed success. Utilizing a combination of bacteriophage and immunomagnetic separation, previous studies have successfully detected one to three CFU per 25 g of food^50^. This method required 20 h to complete but, as a result of the limited host range of the phage (SJ2), could only reliably detect *Salmonella enterica* subsp. *enterica* serovar Enteritidis. Bacteriophage have also been combined with real-time PCR to achieve rapid detection of low burdens in spiked chicken samples^51^. This approach was able to detect eight *Salmonella* CFU per 25 g portion within 10 h. Once again, however, the limited host range of the phage restricted reliable detection to one serovar, Enteritidis. Similar to the approach used in this study, several recombinant luciferase reporter phages have previously been assessed in food matrices. A luciferase-expressing recombinant of the temperate phage SPC32H demonstrated the ability to detect as few as 22 CFU per g (550 CFU per 25 g) in only 2 h from food matrices^49^. Although promising with sensitivity and speed, this phage was specific for the serovar Typhimurium, preventing its use in *Salmonella* species detection. P22, another temperate bacteriophage of *Salmonella*, was also assessed as a luciferase recombinant^20^. This reporter phage demonstrated excellent performance in feed and environmental samples with a 16 h assay time. As with all other described approaches, detection of *Salmonella* by P22 recombinants was limited by the narrow host range of this phage and was serovar dependent. Thus, while previous bacteriophage-based methods have achieved excellent sensitivity and time to results, the PhageDx method uniquely affords broad detection of *Salmonella* independent of serovar in food matrices.

Ultimately, new methods support the continued goal of preventing contaminated products from reaching consumers. Shorter enrichment times are highly desirable, allowing issues to be detected early with limited product holding time. The PhageDx *Salmonella* assay leverages the sensitivity of two engineered NanoLuc-expressing bacteriophages to achieve rapid detection of single cell *Salmonella* contamination. This study demonstrates the noteworthy capabilities of bacteriophage reporter assays to facilitate accurate pathogen detection in a variety of matrices.

## Methods

### Bacterial strains

All bacterial strains used in this study were obtained from the American Type Culture Collection (ATCC) (Manassas, VA), University of Georgia (Athens, GA), University of Iowa (Iowa City, IA), United States Department of Agriculture (USDA) (Clay Center, NE), Michigan State University STEC Center (East Lansing, MI), and the Food and Drug Administration (FDA) (College Park, MD). Unless otherwise indicated, bacterial strains were routinely cultured overnight in tryptone soy broth (TSB) (Oxoid, Hampshire, United Kingdom) at 37 °C with shaking at 225 revolutions per minute (rpm).

### Wild-type phage isolation

The *Salmonella* bacteriophage SEA1 was obtained from Dr. Francisco Diez-Gonzalez’s laboratory at the University of Minnesota. SEA1 is a broad-spectrum *Salmonella* phage of the *Myoviridae* family previously isolated from waste effluents^52^. *Salmonella* phage TSP1 was isolated from sewage samples obtained from the Metropolitan Waste Water Treatment Plant in St. Paul, Minnesota. Samples were clarified by centrifuging in a swinging bucket rotor at 4700 × *g* for 10 min and filtering the supernatant through a 0.45 µm filter (Nalgene, Rochester, NY). A mixture containing 2 mL of this filtrate, 1 mL of 3 × TSB, and 150 µL of an overnight culture of *Salmonella enterica* subsp. *enterica* ser. Typhimurium (ATCC 19585) was incubated at 37 °C with shaking at 225 rpm for 18 h. After this incubation, the sample was once again centrifuged at 4700 × *g* for 10 min and the supernatant filtered through a 0.45 µm filter. The presence of phage was initially evaluated by spot testing on ATCC 19585 and confirmed by plating for single plaques using the classical overlay method^53^. Individual plaques were picked, resuspended in TSB, and subsequentially plated again for single plaques. Single plaque selection was repeated five times to obtain pure, single phage cultures.

### Generation of high titer stocks of wild-type and recombinant bacteriophages

High titer wild-type and recombinant phage stocks were made using broth lysates. To this end, 100 mL of logarithmic (log) phase *Salmonella* cells at an OD_600_ of 0.2 were infected at a multiplicity of infection (MOI) of 0.05. SEA1 and SEA1.NL used strain ATCC 14028 while TSP1 and TSP1.NL used strain ATCC 19585. After allowing 5 min for adsorption, infected cells were diluted into 400 mL of prewarmed TSB and incubated at 37 °C with shaking at 250 rpm until lysis was apparent. The phage lysate was clarified by centrifuging at 14,900 × *g* using a type 19 rotor in an Optima XE-90 ultracentrifuge (Beckman Coulter, Brea, CA) for 10 min at 4 °C. Phages were concentrated by centrifuging again at 14,900 × *g* for 2 h at 4 °C. The phage pellet was resuspended in TMS buffer (50 mM Tris–HCl pH 7.8, 10 mM MgCl_2_, and 300 mM NaCl) then treated with RNase and DNase I. Phages were further purified on a sucrose density gradient (10–30%) in TMS. The phage band was removed and sedimented at 107,200 × *g* using a SW41 Ti rotor in an Optima XE-90 ultracentrifuge for 30 min at 4 °C. Finally, the phage pellet was resuspended in SM buffer (50 mM Tris–HCl pH 7.5, 8 mM MgSO_4_·7H_2_O, 100 mM NaCl, and 0.01% (w/v) gelatin) and the titer determined by serial dilution and plaque counting.

### Bacteriophage characterization

DNA was isolated from the phages and sequenced by Laragen Inc. (Los Angeles, CA) using Illumina MiSeq whole genome sequencing followed by Contig assembly. DNA was isolated by heating 5 × 10^9^ plaque forming units (pfu) at 90 °C for 5 min. DNA was purified from protein by three phenol/chloroform extractions. After removal of phenol/chloroform, 0.1 volume of 3 M sodium acetate and two volumes of ethanol were added to aqueous phase. DNA was precipitated at − 80 °C, pelleted, then washed two times with 70% ethanol. The DNA pellet was dried and then resuspended in deionized water and used for sequencing.

The burst size and replication cycle time of phages were determined using a traditional one-step growth curve on their respective host strains^54^.

Transmission electron microscopy of SEA1 and TSP1 was performed using 400 mesh grids coated with a thin carbon film. Glow discharged grids were floated on cesium chloride density gradient purified phage samples, then stained with 2% uranyl acetate. Images were captured on a Tecnai G2 Spirit BioTWIN at 30 kV.

### Construction of homologous recombinant plasmids

Plasmids were designed to generate NanoLuc-expressing recombinant bacteriophages through homologous recombination (HR). Constructs containing a codon-optimized NanoLuc (Promega Corp., Madison, WI) gene under a T4 late promoter and flanked by regions of homology to the respective phage genome were designed. Codon optimization for *Salmonella* was performed using a codon optimization tool (Integrated DNA Technologies, Coralville, IA). Homologous flanks were designed to direct insertion downstream of the predicated major capsid protein. The SEA1 cassette consisted of 500 bp upstream of the desired insertion site, followed by the σ70 promoter − 10 consensus sequence, a Shine-Dalgarno ribosomal entry site consensus sequence, a NanoLuc codon optimized for *Salmonella*, then 500 bp of downstream phage SEA1 sequence. The TSP1 cassette was similarly designed with the following exception. A 300 bp downstream homologous sequence was used, followed by a stop codon and transcriptional terminator. Differences in design were due to initial difficulties in the construction of the TSP1 HR plasmid which were overcome by the addition of a stop codon and transcriptional terminator. Constructed cassettes targeting TSP1 and SEA1 were assembled and inserted into the multiple cloning site of pUC57 by GeneWiz (South Plainfield, NJ). These recombinant constructs were expected to facilitate insertion of NanoLuc into a phage late gene region without disrupting any predicted genes.

### Integration of the NanoLuc into the phage genome by homologous recombination

Electrocompetent *Salmonella* were generated as described previously^55,56^. *Salmonella enterica* subsp. *enterica* ser. Typhimurium strains ATCC 14028 and ATCC 19585 were selected as recombinant hosts for SEA1 and TSP1, respectively. Electrocompetent bacteria were combined with 100 ng of homologous recombination plasmid DNA and subjected to a 1.8 kV single pulse using a MicroPulser electroporation apparatus (Bio-Rad Laboratories, Hercules, CA). Transformants were isolated following overnight growth on Luria–Bertani (LB) agar containing 100 µg/mL carbenicillin.

Resistant colonies were selected, grown in LB containing 100 µg/mL ampicillin, and infected with SEA1 or TSP1 respectively at various MOIs (0.1–10). Samples were incubated at 37 °C with 220 rpm shaking for 3 h. Following infection, cultures were centrifuged for 2 min at 6800 × *g*. The supernatant was collected, filtered through a 0.45 µm filter, and washed with TMS on a 100 kDa pore protein concentrator PES column (Pierce Biotechnology, Rockford, IL). This was plated as previously described for single plaque isolation. To identify recombinants, candidate plaques were picked, mixed with a diluted overnight culture, and monitored for luciferase expression. Once a NanoLuc-producing isolate had been found for each bacteriophage, it was sequentially passaged at least four times from a single plaque to ensure the stability and purity of the recombinant. After isolation, high titer stocks of SEA1NanoLuc (SEA1.NL) and TSP1NanoLuc (TSP1.NL) were prepared as described previously. Homologous recombination was verified by genome sequencing, as described previously, and confirmed that the desired recombinants had been generated.

### Limit of detection of phages SEA1NanoLuc and TSP1NanoLuc

Log phase *Salmonella* cells (OD_600_ of 0.1–0.5) were diluted in TSB to obtain desired CFU/mL. *Salmonella enterica* subsp. *enterica* ser. Typhimurium (ATCC 19585) was used for TSP1.NL while *Salmonella enterica* subsp. *enterica* ser. Choleraesuis (ATCC 7001) was used for SEA1.NL. 100 µL was then transferred to a 96-well plate and infected with 10 µL of phage reagent (1.2 × 10^7^ pfu/mL in TSB) for 2 h at 37 °C. Luciferase detection solution was prepared as a master mix for each experiment, consisting of 50 µL of NanoGlo buffer, 15 µL Renilla lysis buffer, and 1 µL of NanoGlo substrate (Promega Corp., Madison, WI) per sample well. Following infection, 65 µL of this luciferase detection solution was added to each well and the samples read in a GloMax Navigator luminometer (Promega Corp., Madison, WI) using a 3 min wait time and 1 s integration. Six to ten replicates of each dilution were measured and results averaged. Signal output was relative light units (RLU). Wells containing no *Salmonella* were used to determine background from media, phage, and detection reagents alone.

### Inclusivity and exclusivity of phages SEA1NanoLuc and TSP1NanoLuc

Inclusivity and exclusivity assays were carried out to determine the coverage and specificity of recombinant phages. Overnight stationary phase cultures were diluted in TSB to an OD_600_ of 0.2 (approximately 1.6 × 10^8^ CFU/mL). Aliquots of 100 µL were transferred to 96-well plates and infected with 10 µL of phage reagent. After 2 h of incubation at 37 °C, 65 µL of luciferase detection solution was added to each well. Luminescence was measured as previously described. Positive results were evaluated using a cutoff of 750 RLU.

### Inoculation of raw ground turkey and powdered infant formula (PIF)

Raw ground turkey (85% lean/15% fat Jennie-O, Wilmar, MN) was pre-screened using the PhageDx and culture-based confirmation method described below. Samples of ground turkey were also homogenized and plated on a non-selective agar (tryptone soy agar) to evaluate pre-existing contamination levels. Once the absence of endogenous *Salmonella* had been confirmed, the matrices were inoculated with the indicated *Salmonella enterica* serovars. Strain 27869 (ATCC) and OCT084 (USDA) were used, serovars Newport and Muenster, respectively. A liquid inoculum culture was prepared by transferring a single *Salmonella* colony from a TSB plate into TSB broth and incubating the culture for 18–24 h at 37 °C. Following incubation, the culture was diluted to the target level in buffered peptone water (BPW) (Oxoid, Hampshire, United Kingdom). Inoculums were plated to determine CFU level. Target CFUs levels were an average of 2–20 CFU/mL for low level and 20–100 CFU/mL for high level inoculums. Aliquots of 100 µL of designated inoculum were used to inoculate turkey samples. Based on averaged replicate plating, CFU inoculum per 25 g sample were 1.1 and 1.2 for low level and 11.8 and 8.9 CFU for high level of serovar Newport and Muenster, respectively. Prior to analysis samples were held for 48–72 h post-inoculation at 2–8 °C to allow for equilibration. Low level inoculated samples were expected to yield fractional positive results (25–75% positive), and a high level expected to yield all positive results. Negative control samples were uninoculated. All samples were assessed in a blinded manner as testers were unaware of the inoculum given to each test portion.

For PIF (Up and Up milk-based infant formula with iron, Target, Minneapolis, MN), *Salmonella enterica* was also grown in TSB for 18–24 h at 37 °C. Strain SL476 (FDA) and 52317.1 (USDA) were used, serovars Heidelberg and Reading, respectively. The culture was diluted in BPW, reconstituted in PIF and placed into a speed vacuum for 4–8 h until the sample was completely dried. Contaminated PIF was allowed to equilibrate for 2–4 weeks at room temperature (20–25 °C). After equilibration, an aliquot of dried inoculum was resuspended in 1 mL of BPW and plated to determine CFU level. Using this determined CFU, dried inoculum was then diluted into additional PIF matrix to achieve a low level (1 CFU/100 g) or high level (5 CFU/100 g). Low level inoculated samples were expected to yield fractional positive results (25–75% positive), and a high level expected to yield all positive results. Negative control samples were uninoculated. Samples of PIF were screened prior to inoculation to evaluate pre-existing contamination as described above for ground turkey. As with ground turkey, PIF was also assessed in a blinded manner.

### PhageDx *Salmonella* detection assay for raw ground turkey and PIF

Raw ground turkey (25 g) was placed in a filter bag (Nasco WhirlPak, Fort Atkinson, WI), homogenized, and enriched with pre-warmed (41 °C) BPW in a 1:3 ratio (25 g ground turkey: 75 mL BPW) for 7 h at 41 °C. Powdered infant formula (100 g) was placed in a sample bag with pre-warmed (37 °C) BPW in a 1:3 ratio (100 g PIF: 300 mL BPW) and enriched for 16 h at 37 °C. After enrichment, a 150 µL direct sample for raw ground turkey, or a 150 µL 1:10 diluted sample for PIF, was transferred to a 96-well plate. Volumes of 10 µL of phage reagent were added and samples were incubated at 37 °C for 2 h. Then, 65 µL of luciferase detection solution was added. Luminescence was measured as previously described. Ground turkey samples and powdered infant formula samples with signals ≥ 750 RLUs were considered positive.

### Culture-based confirmation method for *Salmonella*

For raw ground turkey, all samples were culturally confirmed by treating 1 mL of 24 h enriched samples with Dynabeads anti-*Salmonella* (Life Technologies AS, Norway) and then plating beads onto *Salmonella* selective chromogenic plates, CHROMagar *Salmonella* (DRG International, Springfield, NJ). For PIF samples, 100 µL of 24 h enriched samples were plated directly onto *Salmonella* selective chromogenic plates. Plates were incubated for an additional 24 h at 37 °C. The presence of mauve colonies (1–3 mm) indicated a sample positive for *Salmonella*. The 24 h enrichment used in culture-based confirmations was an extended incubation of the samples previously tested with the PhageDx method, allowing for matched comparison.

## Supplementary information

Supplementary Information.

## Data Availability

Unannotated raw genome assemblies of SEA1 and TSP1 are available upon request. All other data generated or analyzed during this study are included in this published article (and its Supplementary information files). Researchers receiving resources generated in this study may be asked to sign a Materials Transfer Agreement that covers potential commercial use.
