# Supplementary material for: Accurate and sensitive detection of Salmonella in foods by engineered bacteriophages
Source: Sci Rep. 2020 Oct 15;10:17463. doi: 10.1038/s41598-020-74587-8 (PMC7567081; doi:10.1038/s41598-020-74587-8)
Supplement: Supplementary file 1 — Supplementary Information. [file 41598_2020_74587_MOESM1_ESM.docx]

**Supplementary Information**

**Title:** “Accurate and Sensitive Detection of *Salmonella* in Foods by Engineered Bacteriophages”

**Authors:** Minh M. Nguyen^1^, Jose Gil^2^, Matthew Brown^3^, Eduardo Cesar Tondo^4^, Nathanyelle Soraya Martins de Aquino^4^, Marcia Eisenberg^3^ and Stephen Erickson^1,*^

^1^Laboratory Corporation of America Holdings, New Brighton, MN 55112

^2^Laboratory Corporation of America Holdings, Los Angeles, CA 90062

^3^Laboratory Corporation of America Holdings, Burlington, NC 27215

^4^Laboratório de Microbiologia e Controle de Alimentos, Instituto de Ciência e Tecnologia de Alimentos, Universidade Federal do Rio Grande do Sul (ICTA / UFRGS), Porto Alegre, RS 91501-970

**^*^**e-mail: erickss@labcorp.com

|  | **Positives / Total** | |  | **Positives / Total** | |
| --- | --- | --- | --- | --- | --- |
| **Serovar** | **SEA1.NL** | **TSP1.NL** | **Serovar** | **SEA1.NL** | **TSP1.NL** |
| Abaetetuba | 1/1 | 0/1 | Meleagridis | 3/3 | 1/3 |
| Abony | 1/1 | 1/1 | Menden | 1/1 | 1/1 |
| Adelaide | 3/3 | 0/3 | Michigan | 1/1 | 1/1 |
| Aequatoria | 1/1 | 0/1 | Minnesota | 1/1 | 0/1 |
| Agona | 6/6 | 6/6 | Mississippi | 1/1 | 0/1 |
| Alachua | 1/1 | 1/1 | Monophasic | 2/2 | 2/2 |
| Amsterdam | 1/1 | 0/1 | Montevideo | 11/11 | 2/11 |
| Anatum | 6/6 | 1/6 | Muenchen | 6/6 | 2/6 |
| Bareilly | 1/1 | 1/1 | Muenster | 2/2 | 0/2 |
| Benfica | 1/1 | 0/1 | Newport | 17/17 | 4/17 |
| Bispebjerg | 1/1 | 1/1 | Ngili | 1/1 | 0/1 |
| Braenderup | 1/1 | 0/1 | Ohio | 1/1 | 0/1 |
| Brandenburg | 1/1 | 1/1 | Oranienburg | 1/1 | 0/1 |
| Bredeney | 1/1 | 0/1 | Panama | 4/4 | 4/4 |
| Breukelen | 1/1 | 0/1 | Paratyphi A | 1/1 | 1/1 |
| Cerro | 3/3 | 0/3 | Paratyphi B | 2/2 | 2/2 |
| Champaign | 1/1 | 0/1 | Paratyphi C | 2/2 | 2/2 |
| Chester | 1/1 | 1/1 | Pomena | 1/1 | 0/1 |
| Choleraesuis | 3/3 | 1/3 | Potsdam | 1/1 | 0/1 |
| Derby | 4/4 | 4/4 | Pullorum | 2/2 | 1/2 |
| Dublin | 8/8 | 5/8 | Reading | 2/2 | 2/2 |
| Eko | 1/1 | 0/1 | Remo | 1/1 | 0/1 |
| Enteritidis | 27/28 | 27/28 | Rubislaw | 1/1 | 0/1 |
| Gallinarum | 1/1 | 1/1 | Saintpaul | 3/3 | 2/3 |
| Give | 2/2 | 0/2 | San Diego | 1/1 | 1/1 |
| Hadar | 3/3 | 0/3 | Schwarzengrund | 1/1 | 1/1 |
| Havana | 2/2 | 0/2 | Senftenberg | 6/6 | 1/6 |
| Heidelberg | 9/9 | 9/9 | Simsbury | 1/1 | 0/1 |
| Hvittingfoss | 1/1 | 1/1 | Stanley | 1/1 | 1/1 |
| Illinois | 1/1 | 0/1 | Taksony | 1/1 | 0/1 |
| Infantis | 5/5 | 2/5 | Tallahassee | 1/1 | 0/1 |
| Javiana | 2/2 | 1/2 | Tennessee | 5/5 | 1/5 |
| Kahla | 1/1 | 0/1 | Thompson | 5/5 | 1/5 |
| Kalamu | 1/1 | 0/1 | Typhi | 1/1 | 1/1 |
| Kentucky | 2/3 | 0/3 | Typhimurium | 29/29 | 28/29 |
| Kiambu | 2/2 | 2/2 | Uganda | 1/1 | 0/1 |
| Lexington | 1/1 | 0/1 | Urbana | 1/1 | 0/1 |
| Liverpool | 1/1 | 0/1 | Vellore | 1/1 | 0/1 |
| Livingstone | 1/1 | 0/1 | Virchow | 1/1 | 0/1 |
| London | 2/2 | 0/2 | Wagadugu | 1/1 | 1/1 |
| Manhattan | 1/1 | 0/1 | Weltevreden | 1/1 | 0/1 |
| Mbandaka | 3/3 | 0/3 | Worthington | 1/1 | 0/1 |
| **Summary:** | | | | 243/245 | 129/245 |

**Supplementary Table 1:** **Reporter inclusivity with individual *Salmonella enterica* subsp. *enterica* serovars.** Stationary phase cultures were diluted to an OD_600_ of 0.2 and infected with indicated reporter phage for 2 h. Strains were determined to be positive when signal exceeded a detection threshold of 750 relative light units (RLU).

| **Species** | **Subsp.** | **Serovar** | **Source** | **Strain ID** | **SEA1.NL** | **TSP1.NL** |
| --- | --- | --- | --- | --- | --- | --- |
| *enterica* | *enterica* | Abaetetuba | ATCC | 35640 | 81,474,472 | 41 |
| *enterica* | *enterica* | Abony | ATCC | BAA-2162 | 13,025,342 | 150,014,544 |
| *enterica* | *enterica* | Adelaide | USDA | 43128 | 2,925,056 | 64 |
| *enterica* | *enterica* | Adelaide | USDA | SEP293 | 1,948,410 | 61 |
| *enterica* | *enterica* | Adelaide | UIA | DMSO08 | 20,620,000 | 25 |
| *enterica* | *enterica* | Aequatoria | USDA | 1345 | 240,941,440 | 173 |
| *enterica* | *enterica* | Agona | FDA | SARB1 | 59,249,952 | 418,878,912 |
| *enterica* | *enterica* | Agona | UGA | SLR 141 | 11,465 | 25,062,894 |
| *enterica* | *enterica* | Agona | UGA | N/A | 1,552,205 | 77,499,008 |
| *enterica* | *enterica* | Agona | USDA | 42113 | 62,850 | 6,195,000 |
| *enterica* | *enterica* | Agona | USDA | SEP054 | 2,958 | 7,694,021 |
| *enterica* | *enterica* | Agona | UIA | DMSO09 | 84,210 | 355,100,000 |
| *enterica* | *enterica* | Alachua | UIA | DMSO12 | 364,253,920 | 295,961,568 |
| *enterica* | *enterica* | Amsterdam | USDA | 41084 | 36,980,000 | 57 |
| *enterica* | *enterica* | Anatum | FDA | SARB2 | 5,719,141,376 | 1,126 |
| *enterica* | *enterica* | Anatum | UGA | SLR 377 | 162,681,744 | 192 |
| *enterica* | *enterica* | Anatum | USDA | 31064.1 | 16,800,000 | 43 |
| *enterica* | *enterica* | Anatum | USDA | NOV091 | 123,844,296 | 42 |
| *enterica* | *enterica* | Anatum | UIA | DMSO13 | 162,770,832 | 62 |
| *enterica* | *enterica* | Anatum | ATCC | 9270 | 44,377 | 37 |
| *enterica* | *enterica* | Bareilly | UGA | 73 | 185,602,848 | 1,535 |
| *enterica* | *enterica* | Benfica | USDA | AUG071 | 455,400 | 56 |
| *enterica* | *enterica* | Bispebjerg | ATCC | 9842 | 159,974,128 | 137,063,104 |
| *enterica* | *enterica* | Braenderup | USDA | 52115 | 81,969 | 32 |
| *enterica* | *enterica* | Brandenburg | USDA | AUG053 | 662,700 | 664,800,000 |
| *enterica* | *enterica* | Bredeney | USDA | 61003.2 | 11,740,000 | 41 |
| *enterica* | *enterica* | Breukelen | ATCC | 15782 | 2,778,540 | 35 |
| *enterica* | *enterica* | Cerro | USDA | 31011.1 | 407,600,160 | 29 |
| *enterica* | *enterica* | Cerro | USDA | DEC021 | 360,353,696 | 370 |
| *enterica* | *enterica* | Cerro | USDA | V2-577.2 | 292,269 | 114 |
| *enterica* | *enterica* | Champaign | ATCC | 700139 | 100,568,304 | 77 |
| *enterica* | *enterica* | Chester | ATCC | 11997 | 4,558,113,280 | 474,715,712 |
| *enterica* | *enterica* | Choleraesuis | ATCC | 12011 | 134,298,560 | 1,547 |
| *enterica* | *enterica* | Choleraesuis | ATCC | 10708 | 170,185,728 | 291 |
| *enterica* | *enterica* | Choleraesuis | ATCC | 7001 | 98,668,592 | 75 |
| *enterica* | *enterica* | Derby | FDA | SARB9 | 12,198 | 1,419 |
| *enterica* | *enterica* | Derby | FDA | SARB10 | 107,414 | 197,188 |
| *enterica* | *enterica* | Derby | FDA | SARB11 | 197,045,648 | 71,512,520 |
| *enterica* | *enterica* | Derby | USDA | 41088.2 | 1,327,000 | 9,945,000 |
| *enterica* | *enterica* | Dublin | FDA | SL477 | 91,407,056 | 645 |
| *enterica* | *enterica* | Dublin | FDA | SARB12 | 5,843,639,296 | 178 |
| *enterica* | *enterica* | Dublin | FDA | SARB13 | 1,522,073 | 130,845,176 |
| *enterica* | *enterica* | Dublin | FDA | SARB14 | 5,853,678,080 | 135,589,440 |
| *enterica* | *enterica* | Dublin | USDA | 63205 | 304,334 | 896 |
| *enterica* | *enterica* | Dublin | USDA | JUL052 | 45,290 | 47 |
| *enterica* | *enterica* | Dublin | UIA | DMSO30 | 754,300 | 416,900,000 |
| *enterica* | *enterica* | Dublin | ATCC | 15480 | 1,740,486 | 20,416,442 |
| *enterica* | *enterica* | Eko | USDA | 33006.2 | 18,190,000 | 111 |
| *enterica* | *enterica* | Enteritidis | FDA | SL1224 | 46,333,504 | 342,584,576 |
| *enterica* | *enterica* | Enteritidis | FDA | SL1301 | 149,998,512 | 279,901,088 |
| *enterica* | *enterica* | Enteritidis | FDA | SL1302 | 73,158,880 | 334,015,808 |
| *enterica* | *enterica* | Enteritidis | FDA | SL1303 | 49,470,164 | 382,513,856 |
| *enterica* | *enterica* | Enteritidis | FDA | SARB16 | 35,758,692 | 840,515 |
| *enterica* | *enterica* | Enteritidis | FDA | SARB17 | 94,282,760 | 309,580,832 |
| *enterica* | *enterica* | Enteritidis | FDA | SARB18 | 163,330,640 | 317,366,144 |
| *enterica* | *enterica* | Enteritidis | FDA | SARB19 | 94,616,696 | 279,196,960 |
| *enterica* | *enterica* | Enteritidis | UGA | 430 | 135,373,664 | 280,150,080 |
| *enterica* | *enterica* | Enteritidis | UGA | 457 | 388,313,360 | 230,051,880 |
| *enterica* | *enterica* | Enteritidis | UGA | 1294 | 419,753,056 | 195,128,248 |
| *enterica* | *enterica* | Enteritidis | UGA | 61697 | 257,343,776 | 175,864,400 |
| *enterica* | *enterica* | Enteritidis | UGA | 14027-J | 126,145,192 | 370,417,920 |
| *enterica* | *enterica* | Enteritidis | UGA | 16037-L | 114,888,112 | 206,576,768 |
| *enterica* | *enterica* | Enteritidis | UGA | 180-88 | 119,358,888 | 358,823,040 |
| *enterica* | *enterica* | Enteritidis | UGA | 565-88 | 153,713,504 | 3,818 |
| *enterica* | *enterica* | Enteritidis | UGA | H2292 | 138,354,640 | 249,213,184 |
| *enterica* | *enterica* | Enteritidis | UGA | H4267 | 193,152,624 | 247,790,912 |
| *enterica* | *enterica* | Enteritidis | UGA | H4638 | 320,765,888 | 419,026,432 |
| *enterica* | *enterica* | Enteritidis | UGA | ME-14 | 114,413,392 | 207,639,888 |
| *enterica* | *enterica* | Enteritidis | UGA | MH45931 | 356,041,792 | 354,058,432 |
| *enterica* | *enterica* | Enteritidis | UGA | S276 | 604,654 | 3,233,180 |
| *enterica* | *enterica* | Enteritidis | UGA | S293 | 15,881,310 | 383,002,720 |
| *enterica* | *enterica* | Enteritidis | UGA | S294 | 9,607,902 | 390,469,472 |
| *enterica* | *enterica* | Enteritidis | UGA | S421 | 19,267,712 | 404,313,408 |
| *enterica* | *enterica* | Enteritidis | UGA | S492 | 219 | 481 |
| *enterica* | *enterica* | Enteritidis | UIA | 4-52-41 | 3,841,278 | 18,726,718 |
| *enterica* | *enterica* | Enteritidis | ATCC | 13076 | 124,696,224 | 411,170,976 |
| *enterica* | *enterica* | Gallinarum | UIA | 4-50-39 | 117,686,904 | 354,388,800 |
| *enterica* | *enterica* | Give | USDA | 63213 | 221,097,088 | 225 |
| *enterica* | *enterica* | Give | ATCC | 9268 | 1,275,440 | 638 |
| *enterica* | *enterica* | Hadar | UGA | 14145-L | 195,416,064 | 64 |
| *enterica* | *enterica* | Hadar | UGA | MH44684 | 11,075,670 | 69 |
| *enterica* | *enterica* | Hadar | UGA | VA07170803 | 208,948,640 | 87 |
| *enterica* | *enterica* | Havana | UGA | 99-109840 | 26,656,554 | 40 |
| *enterica* | *enterica* | Havana | UGA | MH84665 | 166,512,368 | 748 |
| *enterica* | *enterica* | Heidelberg | FDA | SL476 | 153,022,592 | 25,652,106 |
| *enterica* | *enterica* | Heidelberg | FDA | SL486 | 896,572 | 145,342,928 |
| *enterica* | *enterica* | Heidelberg | FDA | SARB23 | 1,088,842 | 131,853,408 |
| *enterica* | *enterica* | Heidelberg | FDA | SARB24 | 8,853,631 | 406,794,480 |
| *enterica* | *enterica* | Heidelberg | UGA | 6316-J | 207,102,224 | 399,253,088 |
| *enterica* | *enterica* | Heidelberg | USDA | 31026.1 | 139,058 | 28,912,046 |
| *enterica* | *enterica* | Heidelberg | UIA | DMSO38 | 146,097,376 | 357,844,448 |
| *enterica* | *enterica* | Heidelberg | ATCC | 8326 | 226,117,904 | 294,694,144 |
| *enterica* | *enterica* | Heidelberg | FDA | SL1225 | 896,115 | 355,300 |
| *enterica* | *enterica* | Hvittingfoss | USDA | 63008.2 | 237,217,104 | 998 |
| *enterica* | *enterica* | Illinois | ATCC | 11646 | 4,224,671,232 | 62 |
| *enterica* | *enterica* | Infantis | UGA | MH95276 | 109,502,112 | 1,939 |
| *enterica* | *enterica* | Infantis | USDA | 31061 | 736,700,032 | 43 |
| *enterica* | *enterica* | Infantis | USDA | JUL301 | 7,549 | 348 |
| *enterica* | *enterica* | Infantis | UIA | DMSO43 | 2,865,999,872 | 1,129 |
| *enterica* | *enterica* | Infantis | ATCC | 51741 | 145,144,336 | 97 |
| *enterica* | *enterica* | Javiana | UGA | 17339-0 | 2,877,672 | 173 |
| *enterica* | *enterica* | Javiana | ATCC | 10721 | 124,645,424 | 7,005,335 |
| *enterica* | *enterica* | Kahla | ATCC | 17980 | 7,296,855,040 | 202 |
| *enterica* | *enterica* | Kalamu | USDA | 63279.2 | 893,600,000 | 54 |
| *enterica* | *enterica* | Kentucky | ATCC | 9263 | 57,531,032 | 46 |
| *enterica* | *enterica* | Kentucky | USDA | 31028 | 1,984,247 | 55 |
| *enterica* | *enterica* | Kentucky | USDA | 1315 | 113 | 47 |
| *enterica* | *enterica* | Kiambu | USDA | 51316 | 2,900,058 | 324,337,344 |
| *enterica* | *enterica* | Kiambu | USDA | DEC174 | 5,626,906 | 352,658,208 |
| *enterica* | *enterica* | Lexington | UGA | 9492-M | 28,256,152 | 53 |
| *enterica* | *enterica* | Liverpool | USDA | AUG365 | 348,709,856 | 56 |
| *enterica* | *enterica* | Livingstone | USDA | 52327.2 | 118,300 | 45 |
| *enterica* | *enterica* | London | USDA | 43290 | 2,763 | 41 |
| *enterica* | *enterica* | London | USDA | JUL218 | 1,378,000 | 48 |
| *enterica* | *enterica* | Manhattan | USDA | 1342 | 1,518,669 | 45 |
| *enterica* | *enterica* | Mbandaka | UGA | 74 | 196,287,272 | 55 |
| *enterica* | *enterica* | Mbandaka | USDA | 42136 | 3,563,000 | 705 |
| *enterica* | *enterica* | Mbandaka | USDA | NOV145 | 257,196 | 43 |
| *enterica* | *enterica* | Meleagridis | UGA | 92 | 145,308,436 | 50 |
| *enterica* | *enterica* | Meleagridis | USDA | 11008.1 | 156,641,760 | 690 |
| *enterica* | *enterica* | Meleagridis | USDA | FEB095 | 256,379,600 | 1,303 |
| *enterica* | *enterica* | Menden | ATCC | 15992 | 3,560,914,176 | 4,494 |
| *enterica* | *enterica* | Michigan | UGA | N/A | 30,269,623 | 1,534 |
| *enterica* | *enterica* | Minnesota | USDA | 52329.1 | 22,570 | 45 |
| *enterica* | *enterica* | Mississippi | UIA | DMSO49 | 3,029,000 | 461 |
| *enterica* | *enterica* | Monophasic | UGA | 103 | 91,616,056 | 284,687,536 |
| *enterica* | *enterica* | Monophasic | UGA | 102 | 140,004,336 | 277,650,400 |
| *enterica* | *enterica* | Montevideo | FDA | SL1317 | 28,373 | 397,032,784 |
| *enterica* | *enterica* | Montevideo | FDA | SARB30 | 8,468,552 | 568 |
| *enterica* | *enterica* | Montevideo | FDA | SARB31 | 68,566,088 | 47 |
| *enterica* | *enterica* | Montevideo | UGA | VA07123001 | 202,937 | 1,181 |
| *enterica* | *enterica* | Montevideo | UGA | VA07171801 | 56,984 | 59 |
| *enterica* | *enterica* | Montevideo | UGA | VA07172202 | 153,583,056 | 40 |
| *enterica* | *enterica* | Montevideo | UGA | VA07172205 | 141,925,920 | 340 |
| *enterica* | *enterica* | Montevideo | UGA | 90 | 173,869,856 | 49 |
| *enterica* | *enterica* | Montevideo | USDA | 31056.1 | 261,277,616 | 402 |
| *enterica* | *enterica* | Montevideo | USDA | AUG369 | 204,514,096 | 80 |
| *enterica* | *enterica* | Montevideo | ATCC | 8387 | 100,979,464 | 71 |
| *enterica* | *enterica* | Muenchen | FDA | SL1314 | 2,438,382,080 | 15,295 |
| *enterica* | *enterica* | Muenchen | FDA | SARB32 | 1,992,084,608 | 48 |
| *enterica* | *enterica* | Muenchen | FDA | SARB33 | 4,619,074,048 | 542 |
| *enterica* | *enterica* | Muenchen | FDA | SARB35 | 138,832,368 | 52 |
| *enterica* | *enterica* | Muenchen | USDA | 63299 | 2,368,000,000 | 857 |
| *enterica* | *enterica* | Muenchen | USDA | OCT080 | 3,567,180 | 46 |
| *enterica* | *enterica* | Muenster | USDA | 31053 | 3,963,531 | 41 |
| *enterica* | *enterica* | Muenster | USDA | OCT084 | 77,933 | 51 |
| *enterica* | *enterica* | Newport | FDA | SL254 | 144,886,768 | 27 |
| *enterica* | *enterica* | Newport | FDA | SL317 | 362,322,688 | 71 |
| *enterica* | *enterica* | Newport | FDA | SARB36 | 2,961,632,512 | 52 |
| *enterica* | *enterica* | Newport | FDA | SARB37 | 5,417,735,680 | 88 |
| *enterica* | *enterica* | Newport | FDA | SARB38 | 1,479,245,952 | 1,179 |
| *enterica* | *enterica* | Newport | UGA | 11590-K | 106,605,958 | 46 |
| *enterica* | *enterica* | Newport | UGA | MH57137 | 273,264 | 245,019 |
| *enterica* | *enterica* | Newport | UGA | 55 | 190,814,768 | 657 |
| *enterica* | *enterica* | Newport | UGA | 57 | 142,928,016 | 27 |
| *enterica* | *enterica* | Newport | UGA | 78 | 134,727,168 | 45 |
| *enterica* | *enterica* | Newport | UGA | 88 | 154,595,584 | 44 |
| *enterica* | *enterica* | Newport | USDA | 63283 | 2,550,000,128 | 53 |
| *enterica* | *enterica* | Newport | USDA | DEC069 | 233,181,760 | 269 |
| *enterica* | *enterica* | Newport | UIA | DMSO55 | 2,304,000,000 | 3,323 |
| *enterica* | *enterica* | Newport | ATCC | 6962 | 4,816,355,328 | 41 |
| *enterica* | *enterica* | Newport | ATCC | 27869 | 85,592,176 | 46 |
| *enterica* | *enterica* | Newport | FDA | SL1223 | 19,064 | 980 |
| *enterica* | *enterica* | Ngili | ATCC | 19127 | 3,135,790,592 | 25 |
| *enterica* | *enterica* | Ohio | USDA | 52307 | 393,667,680 | 526 |
| *enterica* | *enterica* | Oranienburg | ATCC | 9239 | 70,838,972 | 52 |
| *enterica* | *enterica* | Panama | FDA | SARB39 | 400,174 | 303,791,744 |
| *enterica* | *enterica* | Panama | FDA | SARB40 | 5,163,048,448 | 315,158,432 |
| *enterica* | *enterica* | Panama | FDA | SARB41 | 4,836,790,272 | 339,360,064 |
| *enterica* | *enterica* | Panama | ATCC | 7378 | 91,537,192 | 11,462 |
| *enterica* | *enterica* | Paratyphi A | ATCC | 9150 | 608,260 | 174,519,328 |
| *enterica* | *enterica* | Paratyphi B | ATCC | 10719 | 169,020,552 | 67,675,424 |
| *enterica* | *enterica* | Paratyphi B | USDA | SEP358 | 340,476,128 | 334,997,408 |
| *enterica* | *enterica* | Paratyphi C | ATCC | BAA-1714 | 134,794,720 | 1,022,829 |
| *enterica* | *enterica* | Paratyphi C | ATCC | BAA-1715 | 864,868 | 78,991 |
| *enterica* | *enterica* | Pomena | UIA | DMSO63 | 57,010 | 110 |
| *enterica* | *enterica* | Potsdam | ATCC | 25957 | 4,518,902,272 | 27 |
| *enterica* | *enterica* | Pullorum | UIA | SL297 | 5,138,000 | 53 |
| *enterica* | *enterica* | Pullorum | ATCC | 13036 | 81,527,524 | 2,362,583 |
| *enterica* | *enterica* | Reading | USDA | 52317.1 | 84,120 | 18,230 |
| *enterica* | *enterica* | Reading | USDA | SEP245 | 793,160 | 620,200 |
| *enterica* | *enterica* | Remo | USDA | 43164.2 | 3,339,264 | 153 |
| *enterica* | *enterica* | Rubislaw | UIA | DMSO67 | 105,790,272 | 407 |
| *enterica* | *enterica* | Saintpaul | ATCC | 9712 | 82,576,576 | 415,529,792 |
| *enterica* | *enterica* | Saintpaul | FDA | SARB55 | 1,218,853,760 | 81,410,360 |
| *enterica* | *enterica* | Saintpaul | FDA | SARB56 | 26,428,884 | 687 |
| *enterica* | *enterica* | San Diego | USDA | APR025 | 9,522,000 | 103,000,000 |
| *enterica* | *enterica* | Schwarzengrund | USDA | 13092.2 | 7,521,089 | 26,487,490 |
| *enterica* | *enterica* | Senftenberg | ATCC | 43845 | 2,366 | 118 |
| *enterica* | *enterica* | Senftenberg | FDA | SL1315 | 333,627,104 | 41 |
| *enterica* | *enterica* | Senftenberg | USDA | 31072.1 | 23,980,000 | 36 |
| *enterica* | *enterica* | Senftenberg | USDA | SEP160 | 261,634,272 | 52 |
| *enterica* | *enterica* | Senftenberg | FDA | SARB59 | 27,823,494 | 1,282 |
| *enterica* | *enterica* | Senftenberg | UGA | 15106q | 9,993,046 | 35 |
| *enterica* | *enterica* | Simsbury | ATCC | 12004 | 1,540,680,960 | 37 |
| *enterica* | *enterica* | Stanley | ATCC | 7308 | 68,169,976 | 16,221 |
| *enterica* | *enterica* | Taksony | USDA | 32133 | 41,560,000 | 359 |
| *enterica* | *enterica* | Tallahassee | ATCC | 12002 | 2,089,881 | 56 |
| *enterica* | *enterica* | Tennessee | FDA | SL1517 | 157,676,160 | 33 |
| *enterica* | *enterica* | Tennessee | FDA | SL487 | 8,807,423 | 236 |
| *enterica* | *enterica* | Tennessee | FDA | SL490 | 107,866,232 | 439 |
| *enterica* | *enterica* | Tennessee | FDA | SL63 | 4,317,481,984 | 40 |
| *enterica* | *enterica* | Tennessee | FDA | TW880 | 1,870,579,072 | 1,004 |
| *enterica* | *enterica* | Thompson | UGA | 11842M | 137,041,760 | 445 |
| *enterica* | *enterica* | Thompson | UGA | 15371-K | 1,482,658 | 970 |
| *enterica* | *enterica* | Thompson | USDA | 32117 | 1,587,000,064 | 52 |
| *enterica* | *enterica* | Thompson | USDA | DEC142 | 242,732,464 | 510 |
| *enterica* | *enterica* | Thompson | UIA | DMSO76 | 101,072,624 | 69 |
| *enterica* | *enterica* | Typhi | ATCC | 6539 | 3,280,334,592 | 26,008 |
| *enterica* | *enterica* | Typhimurium | USDA | 31049.2 | 52,521,196 | 33 |
| *enterica* | *enterica* | Typhimurium | USDA | AUG247 | 40,871,196 | 16,143 |
| *enterica* | *enterica* | Typhimurium | UIA | SL1344 | 355,395,488 | 292,480,608 |
| *enterica* | *enterica* | Typhimurium | ATCC | 19585 | 310,010,656 | 365,391,456 |
| *enterica* | *enterica* | Typhimurium | FDA | SL1226 | 379,277,792 | 407,384,192 |
| *enterica* | *enterica* | Typhimurium | FDA | SL1279 | 385,405,440 | 342,914,944 |
| *enterica* | *enterica* | Typhimurium | FDA | SL1280 | 132,328,016 | 379,735,136 |
| *enterica* | *enterica* | Typhimurium | FDA | SL1281 | 183,160,000 | 381,709,760 |
| *enterica* | *enterica* | Typhimurium | FDA | SL1282 | 108,731,616 | 406,282,688 |
| *enterica* | *enterica* | Typhimurium | FDA | SL1283 | 126,172,176 | 398,853,888 |
| *enterica* | *enterica* | Typhimurium | FDA | SL1284 | 2,840,402,176 | 362,198,336 |
| *enterica* | *enterica* | Typhimurium | FDA | SL1285 | 80,220,416 | 364,551,952 |
| *enterica* | *enterica* | Typhimurium | FDA | SL1286 | 28,294,146 | 351,644,144 |
| *enterica* | *enterica* | Typhimurium | FDA | SL1287 | 24,037,144 | 356,556,720 |
| *enterica* | *enterica* | Typhimurium | FDA | SL1288 | 18,726,582 | 400,137,712 |
| *enterica* | *enterica* | Typhimurium | FDA | SL1289 | 51,395,736 | 347,941,952 |
| *enterica* | *enterica* | Typhimurium | FDA | SL1290 | 70,877,360 | 352,810,112 |
| *enterica* | *enterica* | Typhimurium | FDA | SL1291 | 203,489,200 | 370,731,456 |
| *enterica* | *enterica* | Typhimurium/DT104 | FDA | SL1292 | 794,836,928 | 151,668,624 |
| *enterica* | *enterica* | Typhimurium/DT104 | FDA | SL1293 | 1,155,311,616 | 409,543,680 |
| *enterica* | *enterica* | Typhimurium | FDA | SARB65 | 2,761,012,224 | 416,909,792 |
| *enterica* | *enterica* | Typhimurium | FDA | SARB66 | 1,850,801,408 | 312,075,760 |
| *enterica* | *enterica* | Typhimurium | FDA | SARB67 | 4,024,307,456 | 244,597,312 |
| *enterica* | *enterica* | Typhimurium | FDA | SARB68 | 2,178,136,320 | 112,672 |
| *enterica* | *enterica* | Typhimurium | UGA | DT104 | 216,010,224 | 418,062,816 |
| *enterica* | *enterica* | Typhimurium | UGA | 9115199 | 101,437,336 | 302,145,952 |
| *enterica* | *enterica* | Typhimurium | UGA | PTC 1 | 92,841,960 | 358,664,448 |
| *enterica* | *enterica* | Typhimurium | FDA | SL1294 | 1,158,475,904 | 419,573,952 |
| *enterica* | *enterica* | Typhimurium/DT104b | FDA | SL1278 | 103,077,872 | 357,650,848 |
| *enterica* | *enterica* | Uganda | USDA | 51278.2 | 4,075,762 | 44 |
| *enterica* | *enterica* | Urbana | ATCC | 9261 | 336,161,120 | 34 |
| *enterica* | *enterica* | Vellore | ATCC | 15611 | 173,703,568 | 533 |
| *enterica* | *enterica* | Virchow | ATCC | 51955 | 3,199,765 | 477 |
| *enterica* | *enterica* | Wagadugu | USDA | 53298 | 3,699,000,064 | 992 |
| *enterica* | *enterica* | Weltevreden | ATCC | BAA-2568 | 8,591,441,920 | 642 |
| *enterica* | *enterica* | Worthington | ATCC | BAA-2085 | 12,412,392 | 40 |
| *enterica* | *arizonae* | - | ATCC | BAA-1577 | 150,926,136 | 1,238,439 |
| *enterica* | *arizonae* | - | ATCC | 33952 | 71,166,208 | 63 |
| *enterica* | *arizonae* | - | ATCC | 29933 | 4,076 | 23 |
| *enterica* | *arizonae* | - | ATCC | BAA-731 | 1,321,403 | 291 |
| *enterica* | *arizonae* | - | ATCC | 13314 | 1,552,442 | 41 |
| *enterica* | *arizonae* | - | ATCC | 12323 | 4,482,480,512 | 429 |
| *enterica* | *diarizonae* | - | ATCC | BAA-216 | 103,257,000 | 2,341 |
| *enterica* | *diarizonae* | - | ATCC | BAA-639 | 6,567,382,528 | 37 |
| *enterica* | *diarizonae* | - | ATCC | 12325 | 7,543,692,800 | 36 |
| *enterica* | *diarizonae* | - | ATCC | 29934 | 5,810,232,832 | 37 |
| *enterica* | *diarizonae* | - | ATCC | 31241 | 6,029,409 | 26 |
| *enterica* | *diarizonae* | - | ATCC | BAA-1579 | 4,268,697,856 | 163 |
| *enterica* | *houtenae* | - | ATCC | 43974 | 3,913,850 | 163 |
| *enterica* | *houtenae* | - | ATCC | BAA-1580 | 108,530,496 | 23 |
| *enterica* | *indica* | - | ATCC | 43976 | 5,238,925,824 | 36 |
| *enterica* | *salamae* | - | ATCC | 6959 | 101,438,248 | 446,117,280 |
| *enterica* | *salamae* | - | ATCC | 700149 | 159,562 | 61 |
| *enterica* | *salamae* | - | ATCC | 700151 | 55,493,530 | 101 |
| *enterica* | *salamae* | - | ATCC | 29931 | 81,193 | 992 |
| *enterica* | *salamae* | - | ATCC | 700148 | 3,965,946,880 | 4,991 |
| *enterica* | *salamae* | - | ATCC | 43972 | 4,703,879 | 62,647,396 |
| *bongori* | - | - | ATCC | 43975 | 51,580 | 60 |
| Nontypeable | - | - | USDA | 63214 | 63,280,000 | 224 |
| Nontypeable | - | - | USDA | 63393 | 1,550,662 | 37 |

**Supplementary Table 2.** **RLU values for *Salmonella* inclusivity of SEA1.NL and TSP1.NL.** Stationary phase cultures were diluted to an OD_600_ of 0.2 and infected for 2 h with indicated reporter phage. If provided by source, *S. enterica* subsp. *enterica* serovar is indicated. Strains were obtained from either the American Type Culture Collection (ATCC), the University of Georgia (UGA), the University of Iowa (UIA), the United States Department of Agriculture (USDA), or the Food and Drug Administration (FDA). Luciferase production was quantified as relative light units (RLU) on a luminometer after the addition of substrate. Grey boxes are used to indicate strains that could not be detected by either reporter, 750 RLU detection threshold.

| **Genus** | **Species** | **Serovar** | **ATCC ID** | **SEA1.NL** | **TSP1.NL** |
| --- | --- | --- | --- | --- | --- |
| *Acinetobacter* | *calcoaceticus* | - | 23055 | 289 | 86 |
| *Citrobacter* | *braakii* | - | 51113 | 17,950 | 62 |
| *Citrobacter* | *freundii* | - | 8090 | 40 | 46 |
| *Citrobacter* | *koseri* | - | 25408 | 57 | 53 |
| *Citrobacter* | *sedlakii* | - | 51493 | 24,005,312 | 1,727,327 |
| *Citrobacter* | *werkmanii* | - | 51114 | 62 | 37 |
| *Citrobacter* | *youngae* | - | 29935 | 606 | 23 |
| *Cronobacter* | *sakazakii* | - | BAA-894 | 128 | 65 |
| *Edwardsiella* | *tarda* | - | 15947 | 119 | 52 |
| *Enterobacter* | *cloacae* | - | 13047 | 53 | 49 |
| *Enterobacter* | *kobei* | - | BAA-260 | 69 | 48 |
| *Escherichia* | *coli* | - | 8739 | 224 | 56 |
| *Escherichia* | *coli* | - | 9637 | 50 | 52 |
| *Escherichia* | *coli* | O1:K1:H7 | 11775 | 67 | 57 |
| *Escherichia* | *coli* | O111:K58(B4):H- | 33780 | 102,500 | 50 |
| *Escherichia* | *coli* | O157:H7 | 35150 | 42 | 35 |
| *Escherichia* | *coli* | - | 35218 | 214 | 67 |
| *Escherichia* | *coli* | O157:H7 | 43888 | 53 | 48 |
| *Escherichia* | *coli* | O157:H7 | 43890 | 62 | 37 |
| *Escherichia* | *coli* | - | 51813 | 74 | 53 |
| *Escherichia* | *coli* | O157:NM | 700377 | 40 | 26 |
| *Escherichia* | *coli* | O121 | BAA-2190 | 90,554 | 48 |
| *Escherichia* | *coli* | O111:H8 | BAA-2201 | 50 | 37 |
| *Escherichia* | *coli* | O145 | BAA-2206 | 56 | 39 |
| *Escherichia* | *coli* | O103:H2 | BAA-2210 | 279 | 98 |
| *Escherichia* | *coli* | O145:H25 | BAA-2211 | 749 | 301 |
| *Escherichia* | *coli* | O145:H34 | BAA-2216 | 22,770,638 | 48 |
| *Escherichia* | *coli* | O45:H10 | BAA-2649 | 379 | 105 |
| *Escherichia* | *coli* | O157:H- | DEC7E^*^ | 56 | 28 |
| *Escherichia* | *coli* | O157:NM | TWO6555^*^ | 37 | 30 |
| *Escherichia* | *coli* | - | 13706 | 25 | NT |
| *Escherichia* | *coli* | O106:H12 | BAA-1431 | 203 | NT |
| *Escherichia* | *coli* | N:26 | ECOR-42^*^ | 719 | NT |
| *Escherichia* | *coli* | ON:HM | ECOR-45^*^ | 89 | NT |
| *Escherichia* | *coli* | OM:H18 | ECOR-47^*^ | 137 | NT |
| *Escherichia* | *coli* | O1:NM | ECOR-35^*^ | 100 | NT |
| *Escherichia* | *coli* | O2:NM | ECOR-61^*^ | 167 | NT |
| *Escherichia* | *coli* | O4:H43 | ECOR-67* | 143 | NT |
| *Escherichia* | *coli* | O7:NM | ECOR-38* | 115 | NT |
| *Escherichia* | *coli* | O25:N | ECOR-51* | 112 | NT |
| *Escherichia* | *coli* | O55:H7 | DEC5E* | 173 | NT |
| *Escherichia* | *coli* | O78:NM | ECOR70* | 181 | NT |
| *Escherichia* | *coli* | O88:NM | ECOR-34* | 106 | NT |
| *Escherichia* | *coli* | O103:H21 | ECOR-30* | 485 | NT |
| *Escherichia* | *coli* | O104:H21 | ECOR-26* | 150 | NT |
| *Escherichia* | *coli* | O106:NM | ECOR17* | 30 | NT |
| *Escherichia* | *coli* | O116:H21 | Jan105* | 114 | NT |
| *Escherichia* | *coli* | O121:H- | ECOR-21* | 153 | NT |
| *Escherichia* | *coli* | O157:H43 | DEC7C* | 318 | NT |
| *Escherichia* | *coli* | O157:H43 | DEC7D* | 296 | NT |
| *Escherichia* | *coli* | O157 non H7 | FSIS 222.3** | 736 | NT |
| *Escherichia* | *coli* | O157 non H7 | FSIS 66** | 113 | NT |
| *Escherichia* | *coli* | - | SF2.1** | 98 | NT |
| *Escherichia* | *coli* | - | SF1** | 404 | NT |
| *Escherichia* | *coli* | - | SF2.2** | 368 | NT |
| *Escherichia* | *coli* | - | SF5** | 158 | NT |
| *Escherichia* | *coli* | - | SF4** | 149 | NT |
| *Escherichia* | *coli* | - | SF7** | 136 | NT |
| *Escherichia* | *coli* | - | SF9** | 390 | NT |
| *Escherichia* | *fergusonii* | - | 35469 | 70 | 150 |
| *Escherichia* | *hermanni* | - | 33650 | 115 | 299 |
| *Hafnia* | *alevi* | - | 13337 | 487 | 69 |
| *Klebsiella* | *aerogenes* | - | 13048 | 319 | 101 |
| *Klebsiella* | *oxytoca* | - | 43165 | 64 | 81 |
| *Klebsiella* | *pneumonia* | - | 4352 | 283 | 66 |
| *Morganella* | *morganii* | - | 25830 | 63 | 73 |
| *Pluralibacter* | *gergoviae* | - | 33028 | 58 | 53 |
| *Proteus* | *vulgaris* | - | 33420 | 37 | 19 |
| *Pseudomonas* | *aeruginosa* | - | 27853 | 109 | 62 |
| *Serratia* | *marcescens* | - | 13880 | 86,123 | 43 |
| *Shigella* | *flexneri* | - | 12022 | 199,355 | 117 |
| *Shigella* | *sonnei* | - | 9290 | 313 | 52 |
| *Yersinia* | *enterocolitica* | - | 23715 | 4,209 | 35 |
| *Bacillus* | *cereus* | - | 14579 | 67 | 81 |
| *Bacillus* | *subtilis* | - | 6051 | 104 | 82 |
| *Enterococcus* | *faecalis* | - | 19433 | 290 | 165 |
| *Enterococcus* | *faecalis* | - | 29212 | 60 | 61 |
| *Enterococcus* | *faecium* | - | 19434 | 74 | 54 |
| *Listeria* | *grayi* | - | 25401 | 64 | 55 |
| *Listeria* | *innocua* | - | 33090 | 504 | 293 |
| *Listeria* | *ivanovii* | - | 19119 | 81 | 60 |
| *Listeria* | *seeligeri* | - | 35967 | 66 | 63 |
| *Listeria* | *welshimeri* | - | 35897 | 55 | 61 |
| *Staphylococcus* | *aureus* | - | 27660 | 429 | 35 |
| *Staphylococcus* | *aureus* | - | 29213 | 85 | 32 |
| *Staphylococcus* | *aureus* | - | BAA-1721 | 35 | 34 |
| *Staphylococcus* | *epidermidis* | - | 14990 | 87 | 452 |
| *Staphylococcus* | *haemolyticus* | - | 29970 | 68 | 89 |
| *Staphylococcus* | *hominis* | - | 27844 | 299 | 120 |
| *Staphylococcus* | *saprophyticus* | - | 15305 | 52 | 44 |

**Supplementary Table 3.** **RLU values for exclusivity of SEA1.NL and TSP1.NL.** Stationary phase cultures were diluted to an OD_600_ of 0.2 and infected for 2 h with the indicated reporter phage. If available from source, the *E. coli* serovar is indicated. Luciferase production was quantified as relative light units (RLU) on a luminometer after the addition of substrate, 750 RLU detection threshold. ^*^ These strains were obtained from the Michigan State University STEC Center. ^**^ These strains were obtained from the USDA. All other strains were obtained from ATCC. Strain and phage combinations that were not tested are indicated as “NT".
